# Supplementary material for: Design and synthesis of Nrf2-derived hydrocarbon stapled peptides for the disruption of protein-DNA-interactions
Source: PLoS One. 2022 Jun 22;17(6):e0267651. doi: 10.1371/journal.pone.0267651 (PMC9216541; doi:10.1371/journal.pone.0267651)
Supplement: S1 File — (DOCX) [file pone.0267651.s001.docx]

Supplementary Information

Design and Synthesis of Nrf2-Derived Hydrocarbon Stapled Peptides for the Disruption of Protein-DNA-Interactions

Bianca Wiedemann^1 +^, Dominic Kamps^1,2 +^, Laura Depta^1+^, Jörn Weisner^1^, Jana Cvetreznik^1^, Stefano Tomassi^3^, Sascha Gentz^4^, Jan-Erik Hoffmann^4^, Matthias P. Müller^1^, Oliver Koch^5^, Leif Dehmelt^1,2^, and Daniel Rauh^1^ *

^1^ Faculty of Chemistry and Chemical Biology, TU Dortmund University and Drug Discovery Hub Dortmund (DDHD), Zentrum für Integrierte Wirkstoffforschung (ZIW), Otto-Hahn-Strasse 4a, 44227 Dortmund, Germany

^2^ Department of Systemic Cell Biology, Max Planck Institute of Molecular Physiology, Otto-Hahn-Straße 11, 44227 Dortmund (Germany)

^3^ Department of Pharmacy, University of Naples “Federico II”, Via D. Montesano 49, 80131 Napoli, (Italy)

^4^ Protein Chemistry Facility, Max Planck Institute of Molecular Physiology, Otto-Hahn-Straße 11, 44227 Dortmund (Germany)

^5^ Institute of Pharmaceutical and Medicinal Chemistry and German Center of Infection Research, Corrensstr. 48, 48149 Münster (Germany)

^+^ These authors contributed equally to this work.

* Corresponding author:

Daniel Rauh

Faculty of Chemistry and Chemical Biology, TU Dortmund University and Drug Discovery Hub Dortmund (DDHD), Zentrum für Integrierte Wirkstoffforschung (ZIW), Otto-Hahn-Strasse 4a, 44227 Dortmund, Germany

+49 (0)231 755 7080

daniel.rauh@tu-dortmund.de

**Abstract**

Misregulation and mutations of the transcription factor Nrf2 are involved in the development of a variety of human diseases. In this study, we employed the technology of stapled peptides to address a protein-DNA-complex and designed a set of Nrf2-based derivatives. Varying the length and position of the hydrocarbon staple, we chose the best peptide for further evaluation in both fixed and living cells. Peptide **4** revealed significant enrichment within the nucleus compared to its linear counterpart **5**, indicating potent binding to DNA. Our studies suggest that these molecules offer an interesting strategy to target activated Nrf2 in cancer cells.

**Table of Contents**

**Figure S1**: Overlay of PDB snk1-based nrf2 homology model on solution nmr structure (PDB 2lz1).

**Figure S2**: CD spectra of peptides **1**-**5**.

**Table S3**: CD-values used for the determination of helicity of peptides **1**-**5**.

**Table S4**: Sequence and ESI-MS measurements of the peptide library.

**Figure S5:** Fluorescence polarization binding studies of peptides **1-6**.

**Figure S6:** Electrophoretic mobility shift assays of peptides **4** and **5** with MARE23.

**Figure S7:** Electrophoretic mobility shift assays of peptides **4** and **5** with MARE23_scrambled.

**Figure S8:** Electrophoretic mobility shift assays of peptides **4** and **5** with DNA with a randomized sequence.

**Figure S9:** Interaction of peptides **4** and **5** with DNA in fixed and permeabilized cells in the presence of a protease inhibitor mixture.

**Synthetic procedure**

**Figure S10.** Mass spectra of peptide Ac-1.

**Figure S11:** Mass spectra of peptide FITC-1.

**Figure S12:** Mass spectra of peptide Ac-2.

**Figure S13:** Mass spectra of peptide FITC-2.

**Figure S14:** Mass spectra of peptide Ac-3.

**Figure S15:** Mass spectra of peptide FITC-3.

**Figure S16:** Mass spectra and HPLC chromatogram of peptide Ac-4.

**Figure S17:** Mass spectra and HPLC chromatogram of peptide FITC-4.

**Figure S18:** Mass spectra and HPLC chromatogram of peptide Ac-5.

**Figure S19:** Mass spectra and HPLC chromatogram of peptide FITC-5.

**Figure S20:** Raw image used to generate Supplementary Figure S5 by adjusting contrast/brightness and cropping.

**Figure S21:** Raw image used to generate Supplementary Figure S6 by adjusting contrast/brightness and cropping.

**Figure S22:** Raw image used to generate Supplementary Figure S7 by adjusting contrast/brightness and cropping.

**Results**

**
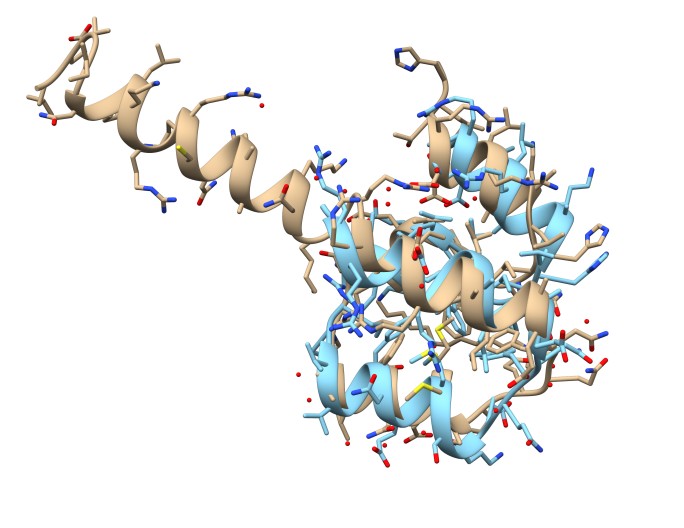
**

**Figure S1**: Overlay of PDB snk1 based Nrf2 homology model on solution (beige) NMR structure (PDB 2lz1, blue).


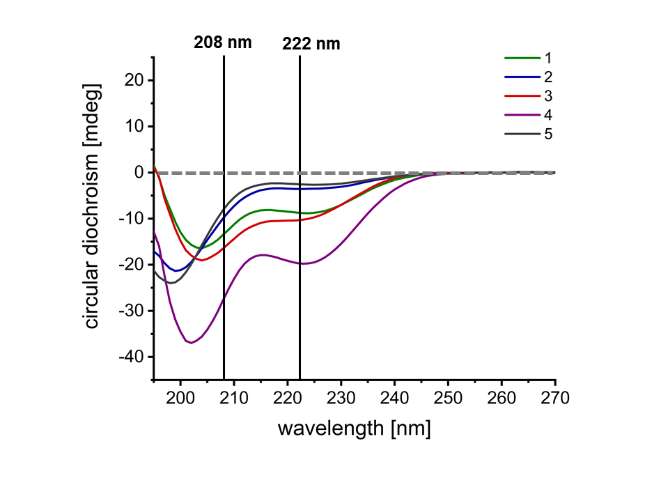


**Figure S2.** CD spectra of peptides **1**-**5**.

**Table S3.** CD-values used for the determination of helicity of peptides 1-5.

| Peptide | Sequence | Θ_Obs_ [mdeg] | Θ_222nm_ [mdeg] | Helicity [%]  (calculated) |
| --- | --- | --- | --- | --- |
| **1** | IRRR**S5**KNK**S5**AAQNCRKRK | 10.3 | -5722 | 20 |
| **2** | **S5**RRR**S5**KNKVAAQNCRKRK | 3.55 | -1972 | 9 |
| **3** | IRRRGKNK**R5**AA**S5**NCRKRK | 19.69 | -10938 | 36 |
| **4** | IRRR**R8**KNKVAA**S5**NCRKRK | 8.7 | -4833 | 18 |
| **5** | IRRRGKNKVAAQNCRKRK | 1.25 | -694 | 6 |

**Table S4.** Sequence and ESI-MS measurements of the peptide library.

|  |  |  |  | ESI-MS [m/z] (calculated) | | | ESI-MS [m/z] (measured) | | | |  |
| --- | --- | --- | --- | --- | --- | --- | --- | --- | --- | --- | --- |
|  | N-term. Modification | Sequence | MW | [M+4H]^4+^ | [M+5H]^5+^ | [M+6H]^6+^ | | [M+4H]^4+^ | [M+5H]^5+^ | [M+6H]^6+^ | yield [mg] |
| **1** | Ac- | RRR**S5**KNK**S5**AAQNCRKRK | 2317.9 | 580.5 | 464.6 | 387.3 | | 580.6 | 464.6 | 387.9 | 4.50  (9 %) |
|  | FITC-GABA- |  | 2637.1 | 660.3 | 528.4 | 440.5 | | 660.2 | 528.6 | 440.6 | 3.60  (7 %) |
| **2** | Ac- | **S5**RRR**S5**KNKVAAQNCRKRK | 2303.8 | 576.9 | 461.8 | 384.9 | | 576.9 | 461.8 | 385.0 | 3.50  (7 %) |
|  | FITC-GABA- |  | 2736.3 | 685.1 | 548.3 | 457.0 | | 685.0 | 548.4 | 457.2 | 2.74  (5 %) |
| **3** | Ac- | IRRRGKNK**R5**AA**S5**NCRKRK | 2246.8 | 562.7 | 450.4 | 375.5 | | 562.7 | 450.5 | 375.5 | 2.75  (6 %) |
|  | FITC-GABA- |  | 2679.2 | 670.8 | 536.8 | 447.5 | | 670.7 | 536.5 | 447.7 | 1.82  (3 %) |
| **4** | Ac- | IRRR**R8**KNKVAA**S5**NCRKRK | 2330.9 | 583.7 | 467.2 | 389.5 | | 583.8 | 467.3 | 389.5 | 4.24  (9 %) |
|  | FITC-GABA- |  | 2763.3 | 691.8 | 553.7 | 461.6 | | 691.8 | 553.6 | - | 4.31  (8 %) |
| **5** | Ac- | IRRRGKNKVAAQNCRKRK | 2223.7 | 556.9 | 445.7 | 371.6 | | 556.9 | 445.8 | 371.7 | 3.67  (8 %) |
|  | FITC-GABA- |  | 2657.1 | 665.3 | 532.4 | 443.9 | | 665.0 | 532.3 | 443.8 | 2.80  (5 %) |
| **6** | Ac- | KNAIQARKCGRKVRRNKR | 2223.7 | 556.9 | 445.7 | 371.6 | | 557.1 | 445.9 | 371.8 | 2.77  (6 %) |
|  | FITC-GABA- |  | 2657.1 | 665.7 | 532.1 | 444.1 | | 665.1 | 532.6 | 443.9 | 4.38  (8 %) |


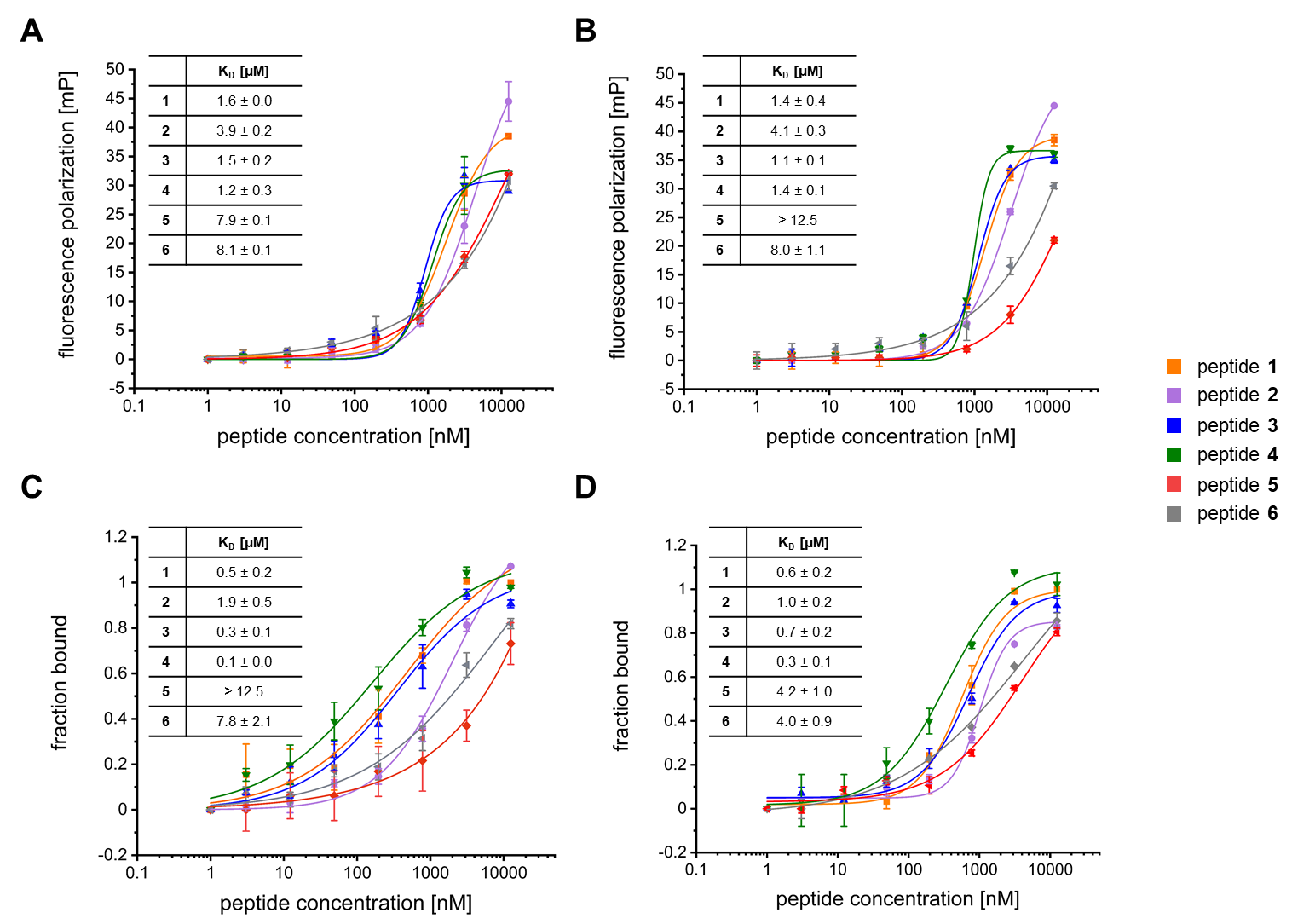


**Figure S5.** Fluorescence polarization binding studies of stapled peptides **1-6** towards target DNA sequence MARE23 (5’‑[6FAM]CGGAATCAATGACTCATTGTTACTC‑3’ and 5’‑GAGTAACAATGAGTCATTGATTCCG‑3’) (**A**, **C**) and control DNA sequence MARE23_scrambled (5’‑[6FAM]CGGAATATCGTCATAGCTATTACTC‑3’ and 5’‑GAGTAA-TAGCTATGACGATATTCCG‑3’) (**B** and **D**, respectively). The obtained polarization values were either baseline-corrected (**A**, **B**) or corrected for the changes in fluorescence intensity in the free/bound state of the fluorescently labeled dsDNA probes (**C**, **D**).^[1]^ The resulting dissociation constants are listed in the inset tables in the respective panels. Error bars indicate the standard deviation with n = 3 independent experiments.


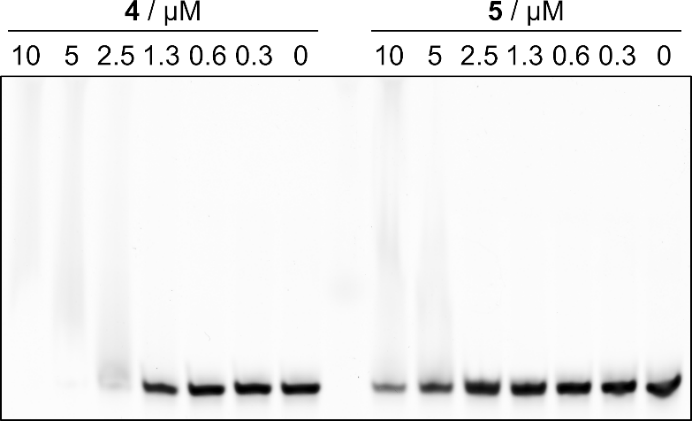
**Figure S6.** Electrophoretic mobility shift assays of peptides **4** and **5** with MARE23 (5’‑[6FAM]CGGAATCAATGACTCATTGTTACTC‑3’ and 5’‑GAGTAACAATGAGTCATTGA-TTCCG‑3’).


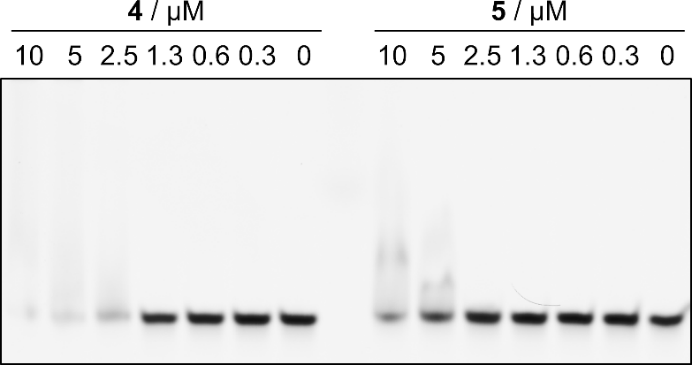


**Figure S7.** Electrophoretic mobility shift assays of peptides **4** and **5** with MARE23_scrambled (5’‑[6FAM]CGGAATATCGTCATAGCTATTACTC‑3’ and 5’‑GAGTAATAGCTATGACGATA-TTCCG‑3’).


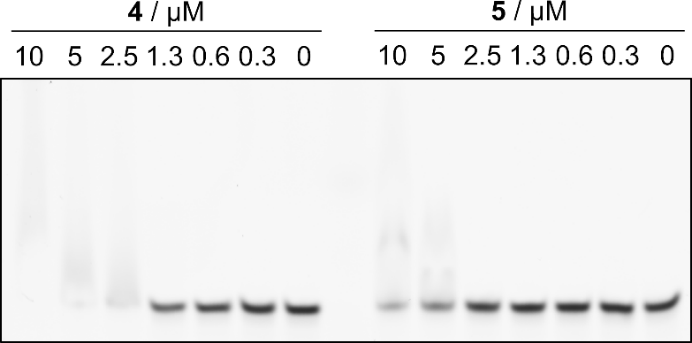


**Figure S8.** Electrophoretic mobility shift assays of peptides **4** and **5** with DNA with a randomized sequence (5’‑[6FAM]CGGAATGCAATAAGTTACGTTACTC‑3’ and 5’‑GAG-TAACGTAACTTATTGCATTCCG‑3’).

**
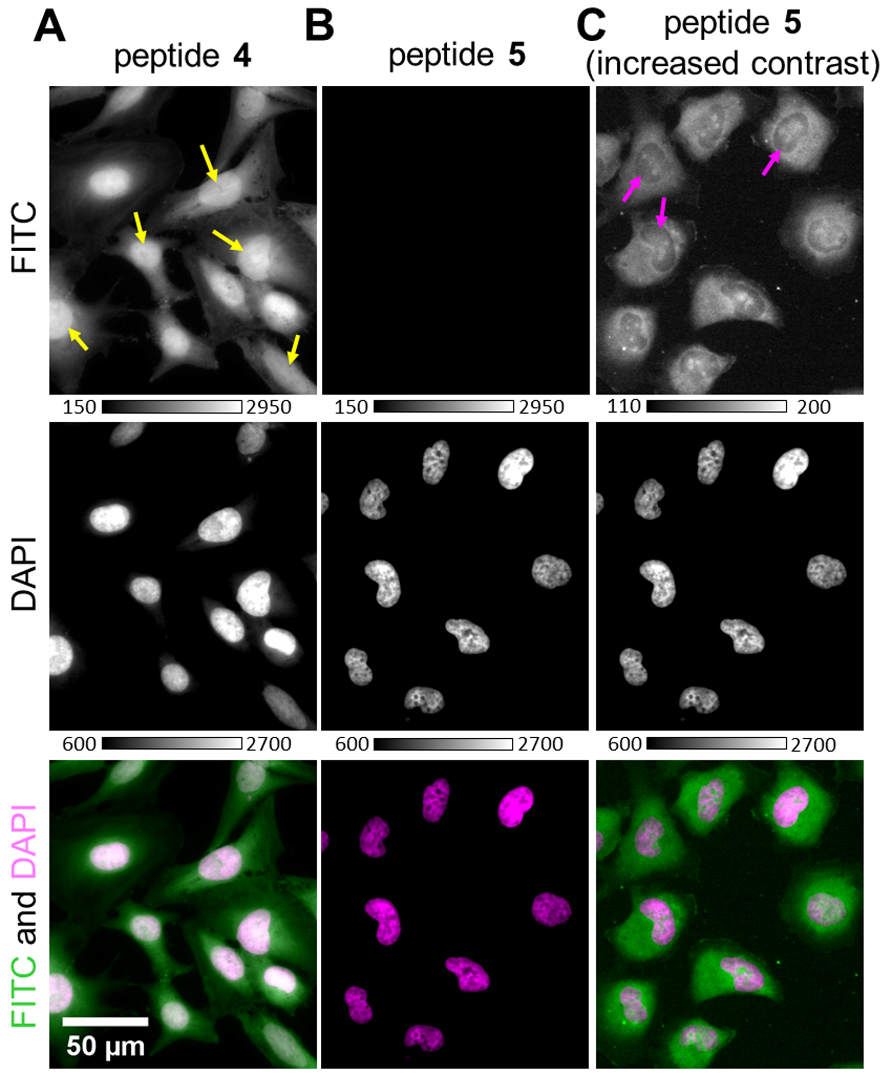
**

**Figure S9.** Enrichment of peptide **4** in DNA-rich nuclei in the presence of a protease inhibitor mixture. **A**-**C**: Fixed and permeabilized HeLa cells were treated with protease inhibitor mix prior to incubation with peptides **4** and **5** and imaged using wide-field fluorescence microscopy. Representative images of FITC-labeled peptides (top panels), DAPI (middle panels), and the combined images (bottom panels) are shown. **A**, **B**: Images showing peptides **4** and **5**, respectively, with identical exposure times and image scaling. **C**: Images of peptide **5** with 30-fold increased image contrast to visualize weak fluorescence of FITC signals.

**Synthetic procedure**

**Resin preparation.**

Rink Amide MBHA LL resin (0.38 mmol/g) was weighed in a column, mixed with 4 mL DMF, and swollen for 45 min with shaking.

**Fmoc cleavage.**

To the resin present in DMF, 25% (v/v) piperidine solution was added in DMF and incubated twice for 15 min with shaking. The resin was then washed with DCM (3 x 4 mL) and DMF (3 x 4 mL).

**Coupling N-terminal-Fmoc protected AS.**

To the resin deprotected as described, a solution of the aa (4 eq.), PyClock (4 eq.), DIPEA (4-8 eq.) and DMF was added and reacted for 120 min at rt.

**Coupling N-terminal-Fmoc protected α-methyl-α-alkenyl modified AS.**

To the resin deprotected as described, a solution of the N-terminal-Fmoc protected α-methyl-α-alkenyl modified aa (3 eq.), PyClock (3-4 eq.), DIPEA (4-6 eq.) and DMF was added and reacted for 120 min at rt.

**Olefin ring closure metathesis (RCM).**

For RCM, the Fmoc-protected resin was first dried under vacuum and then swollen in DCE for 45 min. Then, the resin was run with a solution of dry DCE and Grubbs catalyst (1st generation) (6 mg, 10 mol%) at rt in argon atmosphere to remove ethene formed during the reaction from the reaction mixture. Work was carried out in the absence of light since the Grubbs catalyst (1st generation) is photosensitive. Three cycles, each lasting 1 h, were performed. Finally, washing was performed with DCE (3 x 4 mL), DMF (3 x 4 mL), and DCM (3 x 4 mL).

**Modification of the N-terminus with FITC on the solid phase.**

For modification of the N-terminus with fluorescein, the N-terminal protecting group was first cleaved. Then a solution of fluorescein thioisocyanate (7 eq.), DIPEA (7 eq.) and DMF was added. The reaction was carried out at rt overnight in the absence of light.

**Modification of the N-terminus with an acetyl group at the solid phase.**

For acetylation of the N-terminus, the N-terminal protecting group was cleaved off. Then, a solution of 85 μL acetic anhydride, 315 μL DIPEA, and 1.6 mL DMF was added. The reaction was carried out for 45 min at rt.

**Cleavage of the peptide from the resin.**

For the final cleavage of the peptide from the resin, the resin was dried and transferred completely to a screw cap vial. The resin was stirred for 2.5 h in 2.5 mL of a TFA/TIS solution (20:1). The solution was then filtered through a column to separate the resin. It was washed three times with 5 mL of DCM. The solution was evaporated to a residual volume of 1 mL. Precipitation of the peptide was performed with cold Et_2_O followed by centrifugation at 3200 rpm at 4 °C for 10 min. This procedure was performed twice. The Et_2_O was decanted and the peptide was dried overnight.

**HPLC mediated purification.**

The purification of the peptides was performed by HPLC. The solid was dissolved in a mixture of MeCN:H2O (1:1) and added to the column. A linear gradient was used for elution (1 % MeCN up to 100 % MeCN). The peptide was then lyophilized.


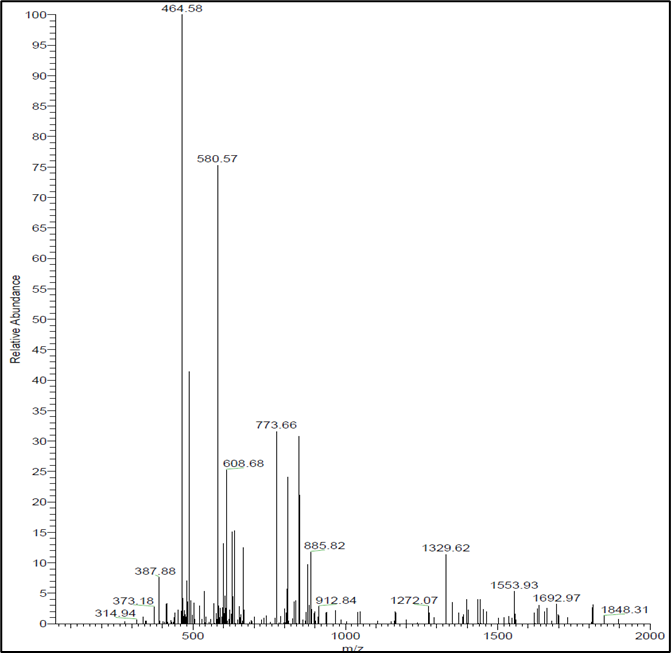


**Figure S10. Mass spectra of peptide Ac-1.**


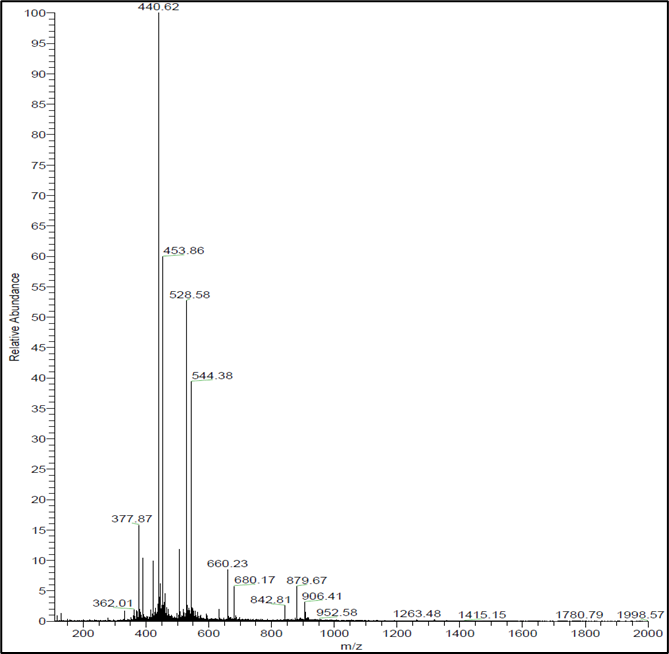


**Figure S11: Mass spectra of peptide FITC-1.**


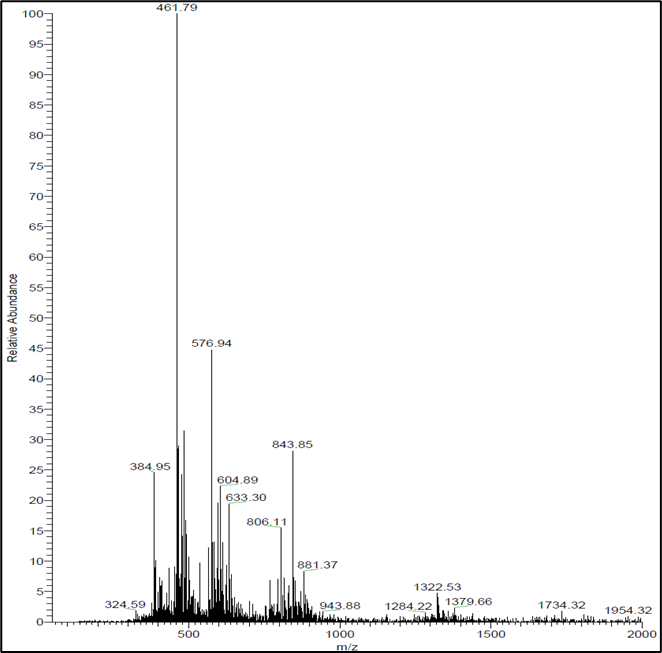


**Figure S12: Mass spectra of peptide Ac-2.**


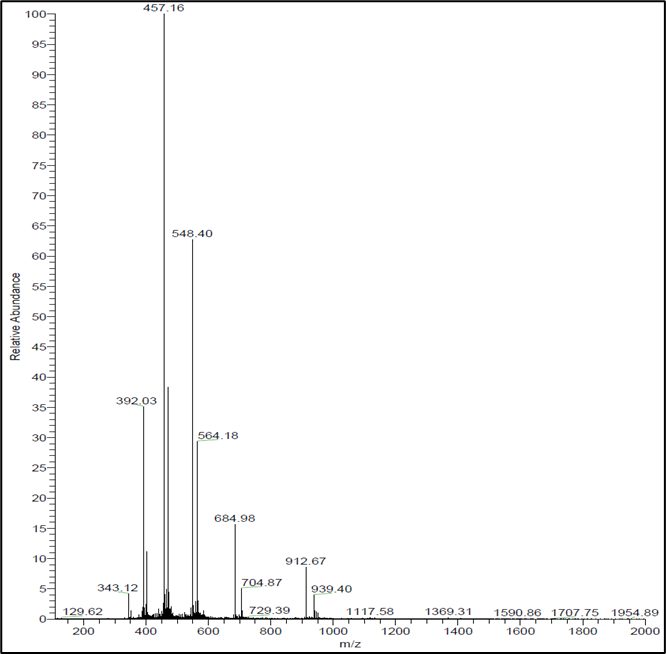


**Figure S13: Mass spectra of peptide FITC-2.**


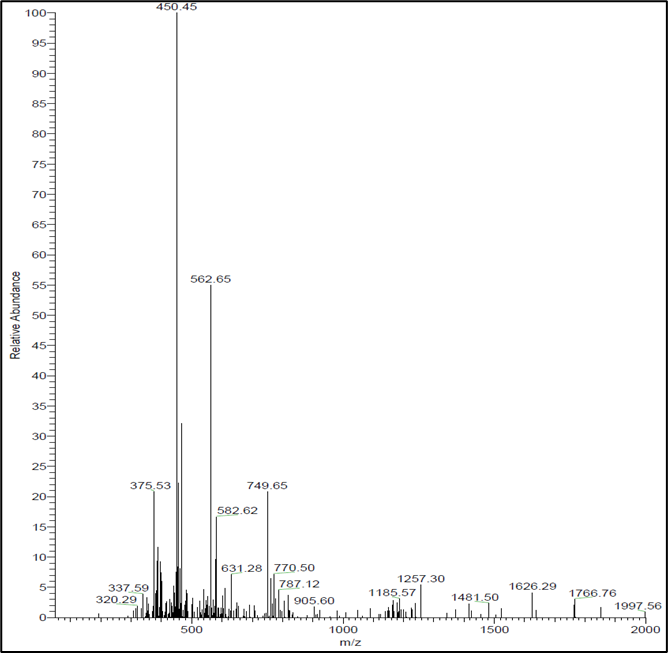


**Figure S14: Mass spectra of peptide Ac-3.**


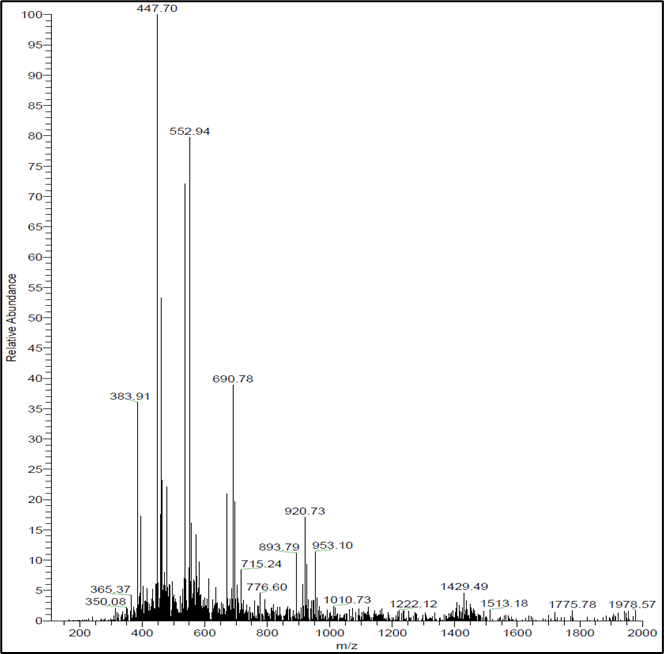


**Figure S15: Mass spectra of peptide FITC-3.**


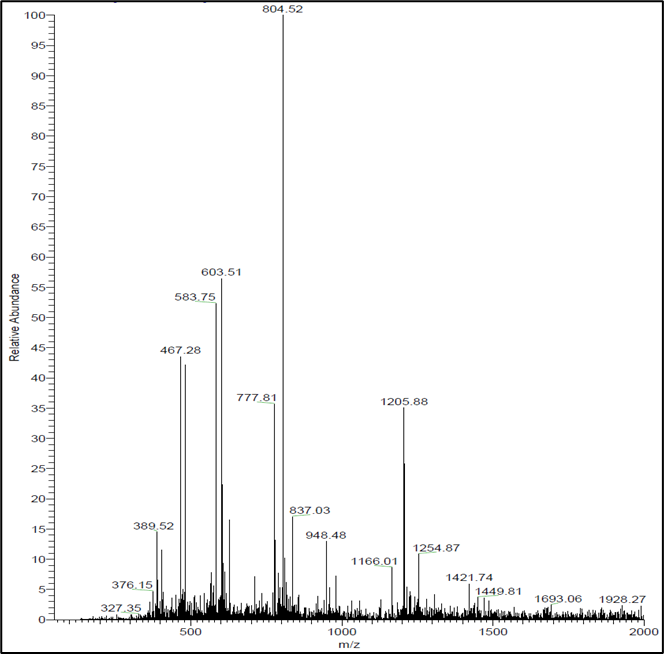


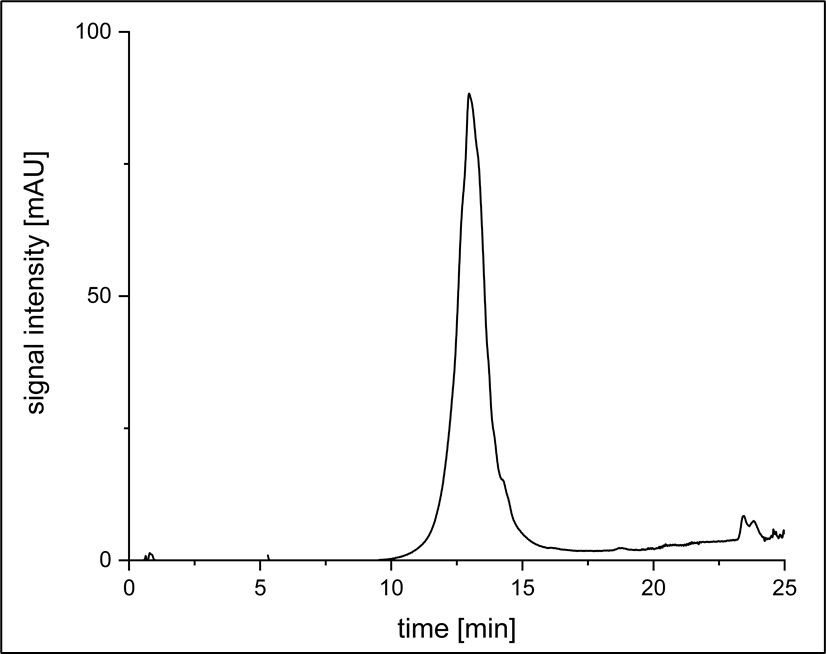


**Figure S16: Mass spectra and HPLC chromatogram of peptide Ac-4.**


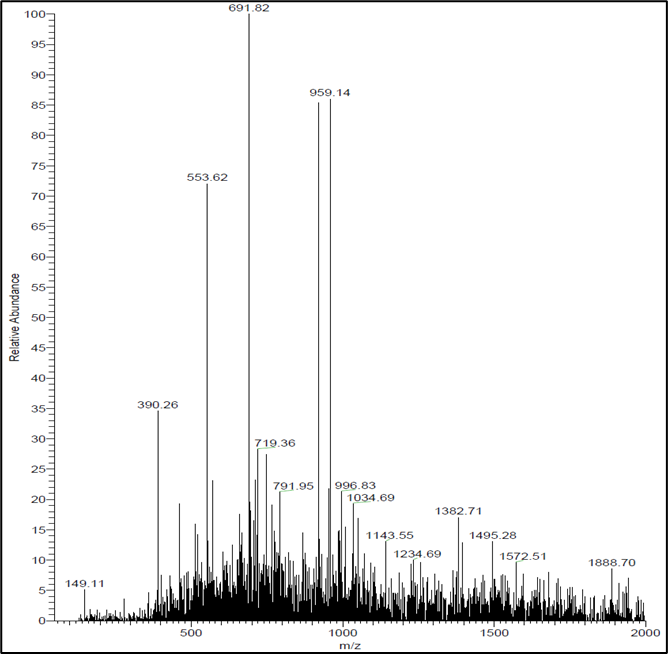


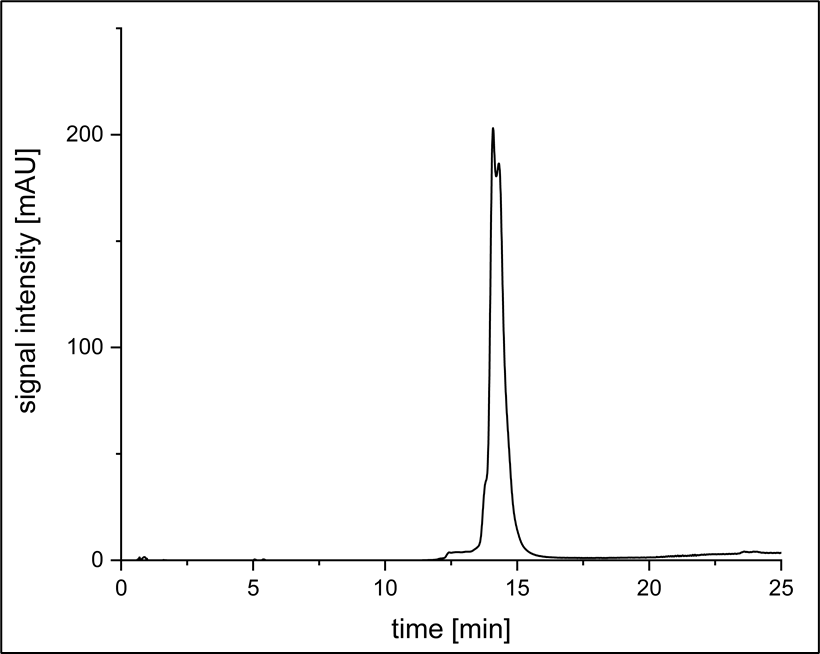


**Figure S17: Mass spectra and HPLC chromatogram of peptide FITC-4.**


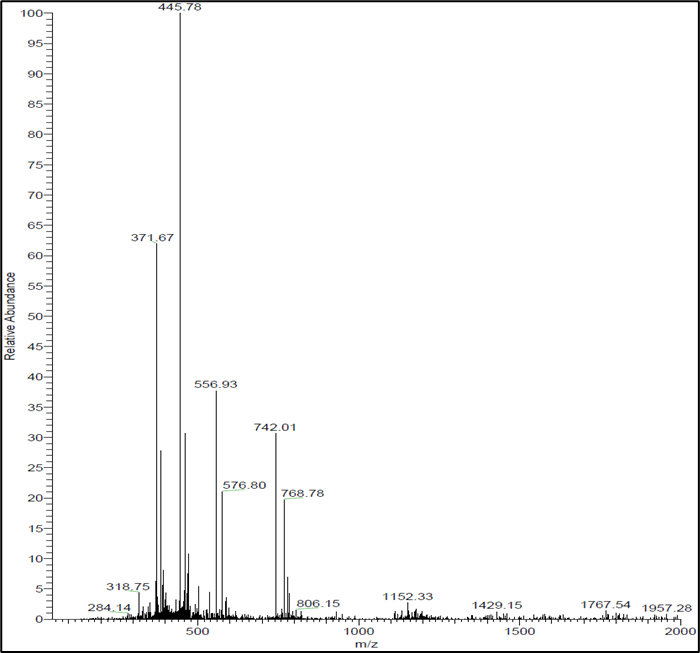


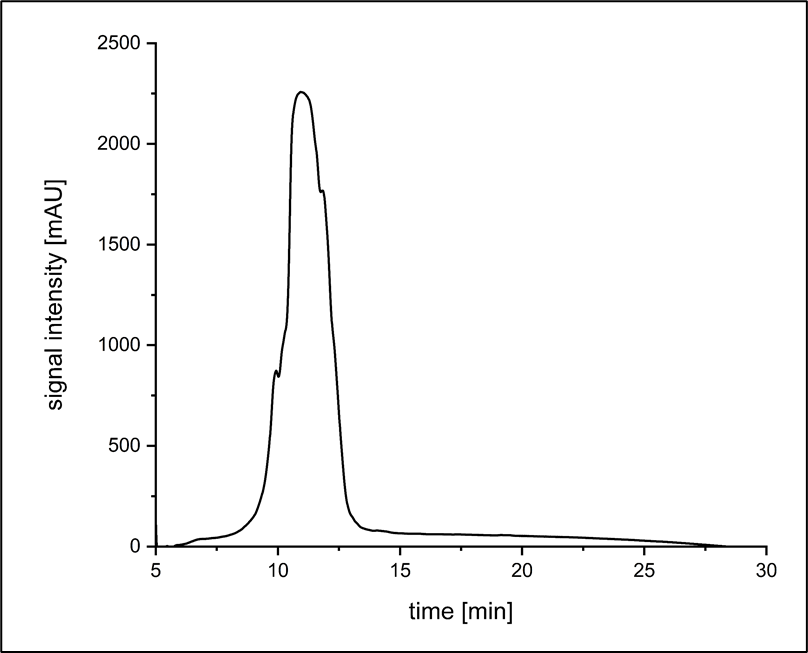


**Figure S18: Mass spectra and HPLC chromatogram of peptide Ac-5.**


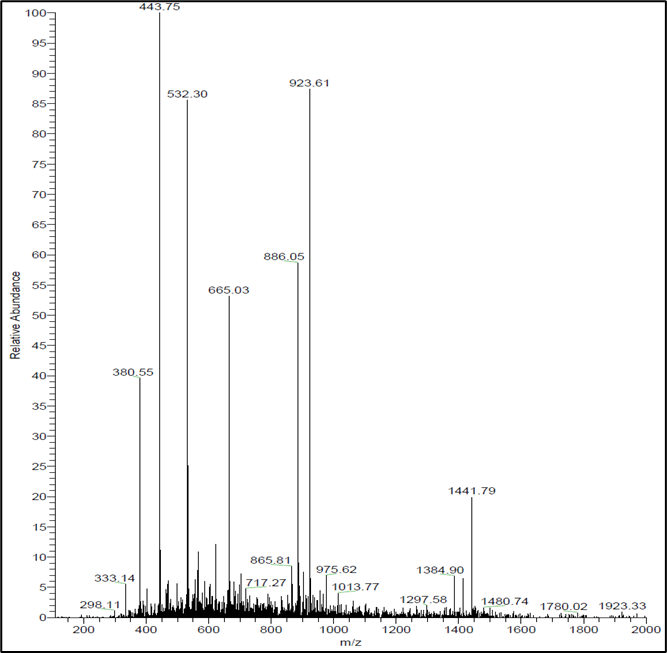


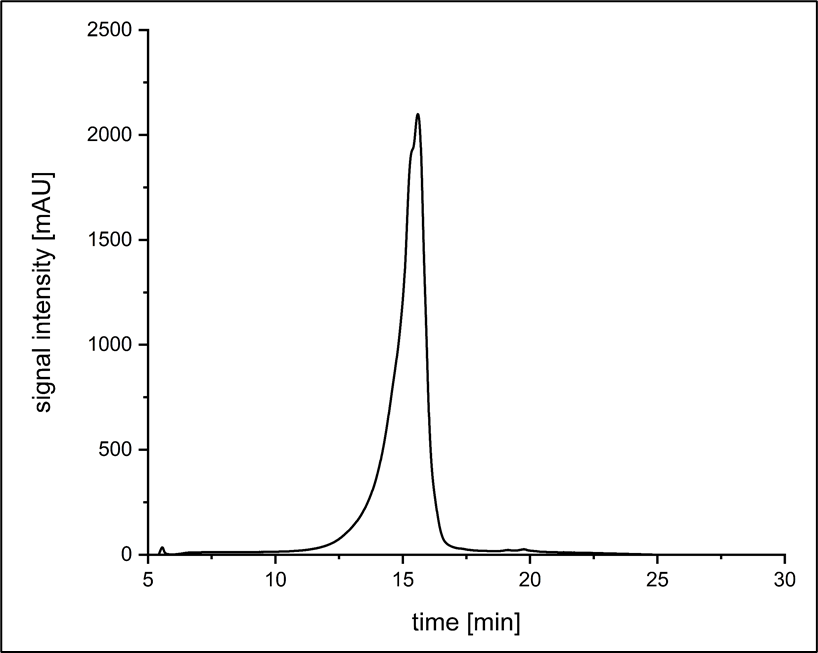


**Figure S19: Mass spectra and HPLC chromatogram of peptide FITC-5.**
