# Supplementary material for: Design and synthesis of Nrf2-derived hydrocarbon stapled peptides for the disruption of protein-DNA-interactions
Source: PLoS One. 2022 Jun 22;17(6):e0267651. doi: 10.1371/journal.pone.0267651 (PMC9216541; doi:10.1371/journal.pone.0267651)
Supplement: S1 Raw images — (PDF) [file pone.0267651.s002.pdf]

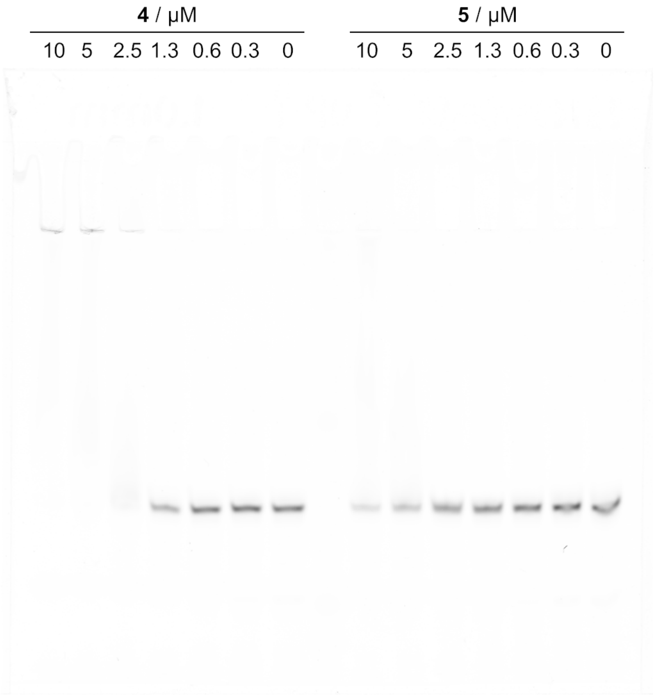

Raw image used to generate Supplementary Figure S5 by adjusting contrast/brightness and cropping.

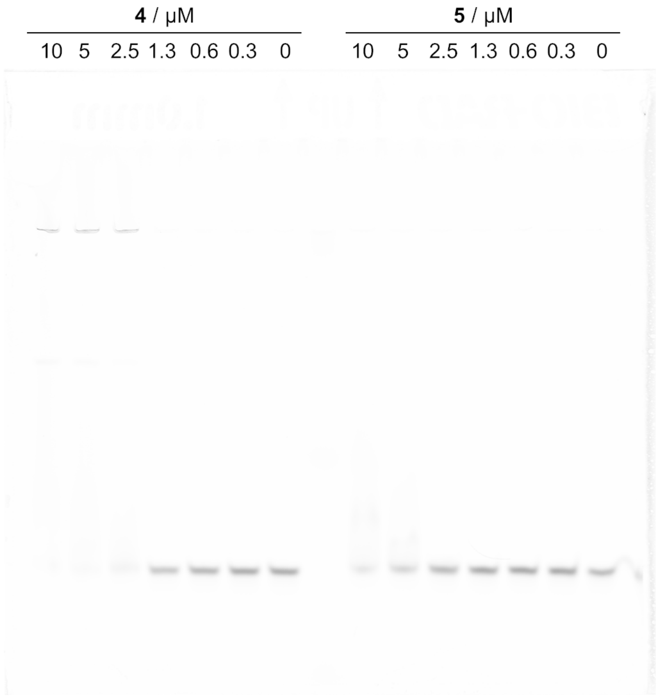

Raw image used to generate Supplementary Figure S6 by adjusting contrast/brightness and cropping.

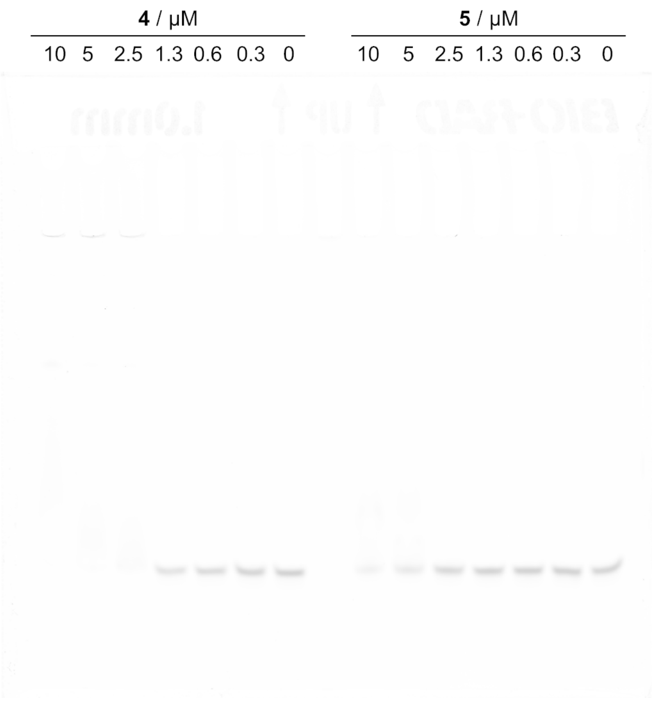

Raw image used to generate Supplementary Figure S7 by adjusting contrast/brightness and cropping.
