# Supplementary material for: Design and synthesis of Nrf2-derived hydrocarbon stapled peptides for the disruption of protein-DNA-interactions
Source: PLoS One. 2022 Jun 22;17(6):e0267651. doi: 10.1371/journal.pone.0267651 (PMC9216541; doi:10.1371/journal.pone.0267651)
Supplement: Attachment — Submitted filename: Response to Reviewers_Wiedemann et al._PLOS ONE.docx [file pone.0267651.s003.docx]

**Response to Decision Letter: Wiedemann *et al*.**

**[PONE-D-22-01699]**

**Editor’s suggestions**

1. Could the authors give more details about the homology modeling procedure in the main text (now is reported in the SI)? It is a critical part of the work as it has inspired the design and synthesis of the peptide library.

**We agree that the homology model is an essential part of our presented work. We now include the homology modeling procedure in the main text (line 103-111).**

2. The authors commented on the flexibility of their approach which could lead to agents able to interfere with RNA activity. I agree with their vision, but I suggest them to also mention the potential selectivity issues emerging from the direct targeting of nucleic acids with peptides.

**We thank the editor for this comment. We now address the selectivity issues in the conclusion.**

**The text reads:”** **However, due to the overall negative charge of nucleic acids, great care must be taken to ensure that nonspecific electrostatic interactions do not interfere with the desired specific binding, requiring careful design and evaluation of the stapled peptide library.“**

**Reviewer #1:**

The authors describe the design, synthesis and testing of stapled peptides meant to target a specific DNA sequence. The authors show convincingly that the peptides bind to DNA, albeit they are not specific to the targeted sequence but bind to other DNA sequences equally well. The experiments are generally described and carried out well, thus the results are well worth publishing in PLOS one.

**We are very grateful and thank the reviewer for the positive assessment of our manuscript.**

1. The authors treat cells with a linear peptide and a stapled peptide. For the latter one, enrichment in the nucleus was observed and therefore the authors concluded that the enrichment is due to stapling. However, both peptides were used at the same concentration, even so the binding affinity for the stapled peptide for DNA is about 6-fold higher than for the linear one. The authors should therefore discuss if the observed enrichment in the nucleus could be rather driven by affinity than the stapling itself.

**We thank the reviewer for the comment and agree that the wording needs to be more precise to clarify this. What we meant to say is that stapling increases the affinity of the peptide and thereby leads to stronger binding and enrichment in the nucleus.**

**The manuscript now reads:”** **In contrast, only small amounts of linear peptide FITC-5 were detectable in cells and no enrichment in the nucleus could be observed for peptide FITC-5, suggesting that nuclear enrichment of the peptides is dependent on stapling and the resulting higher affinity towards DNA.”**

2. The result and discussion section ends rather sudden. In the last sentence, it says that the authors decided to embark in a second round of optimization. However, the optimization is not described in the paper. Is there something lacking in the manuscript or is the second round of optimization a future project?

**This point is well taken. We will** **focus on another round of improved stapled peptides guided by our presented design and results in the future.**

**To clarify this, we rephrased the sentence:” Based on these findings, we will focus on another round of improved stapled peptides guided by our presented design and results in the future, maintaining the stapling system featured by peptide 4 (i,i+7) and focusing on the modification of aa of the aforementioned group c) (see above).**

3. Page 7, line 148, “DNA hybridization was performed as described previously”. Is there are reference lacking or do the authors rather mean “above” instead of “previously”?

**We corrected for this. The text now reads:” DNA hybridization was performed as described above.”**

4. Page 10, line 216, this should read “… is able to induce a mobility shift”

**We corrected for this.**

5. Next line: “… at a lower concentration in respect to the unstapled peptide 5 ..”. This is unclear. Should this read “… at a lower concentration than the unstapled peptide 5 …”?

**We corrected for this. The sentence now reads: “Additionally, the stapled peptide 4 is able to induce a mobility shift of DNA at a lower concentration than the unstapled peptide 5 (Fig. S6‑8).”**

6. Figure S1: Which colour has the model and which colour the NMR structure?

**We added the colours to make this more clear.**
